# Unfolding the Complete Chloroplast Genome of *Myrica esculenta* Buch.‐Ham. ex D.Don (1825): Advancing Phylogenetic Insights Within Fagales and Pioneering DNA Barcodes for Precise Species Identification

**DOI:** 10.1002/ece3.71566

**Published:** 2025-06-12

**Authors:** Raju Balaji, Subbalakshmi Easwaran, Krishnamoorthy Devanathan, Swati Sharma, Taha Alqahtani, Daniel E. Uti, Tabarak Malik

**Affiliations:** ^1^ Department of Orthopedics Saveetha Medical College and Hospital, Saveetha Institute of Medical and Technical Sciences (SIMATS), Saveetha University Chennai Tamil Nadu India; ^2^ Department of Microbiology Saveetha Institute of Medical and Technical Sciences (SIMATS), Saveetha University Chennai Tamil Nadu India; ^3^ Plant Biology and Systematics Lab CSIR‐Central Institute of Medicinal and Aromatic Plants (CIMAP), Research Centre Bengaluru India; ^4^ Department of Pharmacy Chandigarh Pharmacy College, Chandigarh Group of Colleges‐Jhanjeri Mohali Punjab India; ^5^ Department of Pharmacology, College of Pharmacy King Khalid University Abha Saudi Arabia; ^6^ Department of Biochemistry/Research and Publications Kampala International University Kampala Uganda; ^7^ Department of Biochemistry, Faculty of Basic Medical Sciences College of Medicine, Federal University of Health Sciences Otukpo Benue Nigeria; ^8^ Department of Biomedical Sciences, Institute of Health Jimma University Jimma Ethiopia

**Keywords:** chloroplast genome, Fagales, *Myrica esculenta*, *Myrica* species, Myricaceae, phylogenomics, *ycf1*

## Abstract

This study aims to delineate the chloroplast (cp) genome of *Myrica esculenta* Buch.‐Ham. ex D.Don (1825), a traditional medicinal plant from the Myricaceae family, to elucidate its phylogenetic relationships within the Fagales order. The objective was to assemble the complete cp genome and assess its utility as a molecular marker for species identification and evolutionary analysis. The methodology involved assemby of the cp genome of *M. esculenta* , which was found to be 159,538 base pairs (bp) in length and exhibited a typical quadripartite structure. This included an 88,830 bp large single‐copy (LSC) region, an 18,810 bp small single‐copy (SSC) region, and two inverted repeats each of 25,949 bp. Phylogenetic analysis utilized the *ycf1* gene sequences from 13 Fagales species. Results indicated that *M. esculenta* and other *Myrica* species form a monophyletic clade, with the *ycf1* gene showing substantial divergence, suggesting its potential as a novel DNA barcode marker. This marker could significantly improve the resolution of species identification beyond traditional morphological methods. Future perspectives include expanding the genomic datasets across the *Myrica* genus to enhance the phylogenetic framework and further refine the utility of the *ycf1* gene as a DNA barcode for broader applications in plant breeding, herbal drug authentication, and evolutionary studies.

## Graphical abstract

This study presents the complete chloroplast genome of *Myrica esculenta* (159,538 bp) and its phylogenetic placement within the Fagales order. Phylogenetic analysis using the *ycf1* gene confirmed a monophyletic clade with other *Morella* species, highlighting *ycf1* as a promising DNA barcode for species identification. The findings enhance phylogenetic insights and support applications in plant breeding, herbal drug authentication, and evolutionary studies.

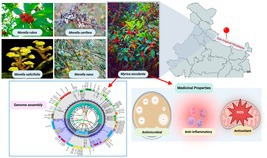

## 1 Introduction

The family Myricaceae, as delineated by Rich. ex Kunth, comprises three recognized genera, including the prominent genus *Myrica* L., recently synonymized under *Myrica*, which encompasses around 50 accepted species. In this study, we adopt *Myrica* as the accepted genus name following Plants of the World Online (POWO), though some literature refers to *Myrica* as a synonym (POWO 2024). Notably, the species *Myrica esculenta*, commonly referred to as Bay Berry, is an evergreen tree indigenous to the hills of Uttarakhand, Western Nepal, and the Eastern Himalayas. This species is used for its myriad uses in traditional medicinal practices such as Ayurveda and Siddha, primarily for its potent antioxidant properties (Shri et al. 2018). Despite its significance, it faces issues of misidentification, often being confused with *Careya arborea*, which complicates its collection for Ayurvedic medicine (Anonymous 2007).

Historically, the order Fagales was restricted to only the Betulaceae and Fagaceae families prior to the 1990s (Takhtajan 1980; Cronquist 1988). However, subsequent phylogenetic studies leveraging DNA sequences and *cpDNA* restriction site analysis have expanded this order to include seven families: Nothofagaceae, Fagaceae, Myricaceae, Juglandaceae (inclusive of Rhoipteleaceae), Casuarinaceae, Ticodendraceae, and Betulaceae (Manos et al. 1993; Chen et al. 2016; APG III 2009; APG IV 2016). While most familial and inter‐familial relationships within the Fagales are well understood, the specific phylogenetic placement of Myricaceae and its associated genera, such as *Myrica*, remains an area of active research and debate.

**FIGURE 1:**
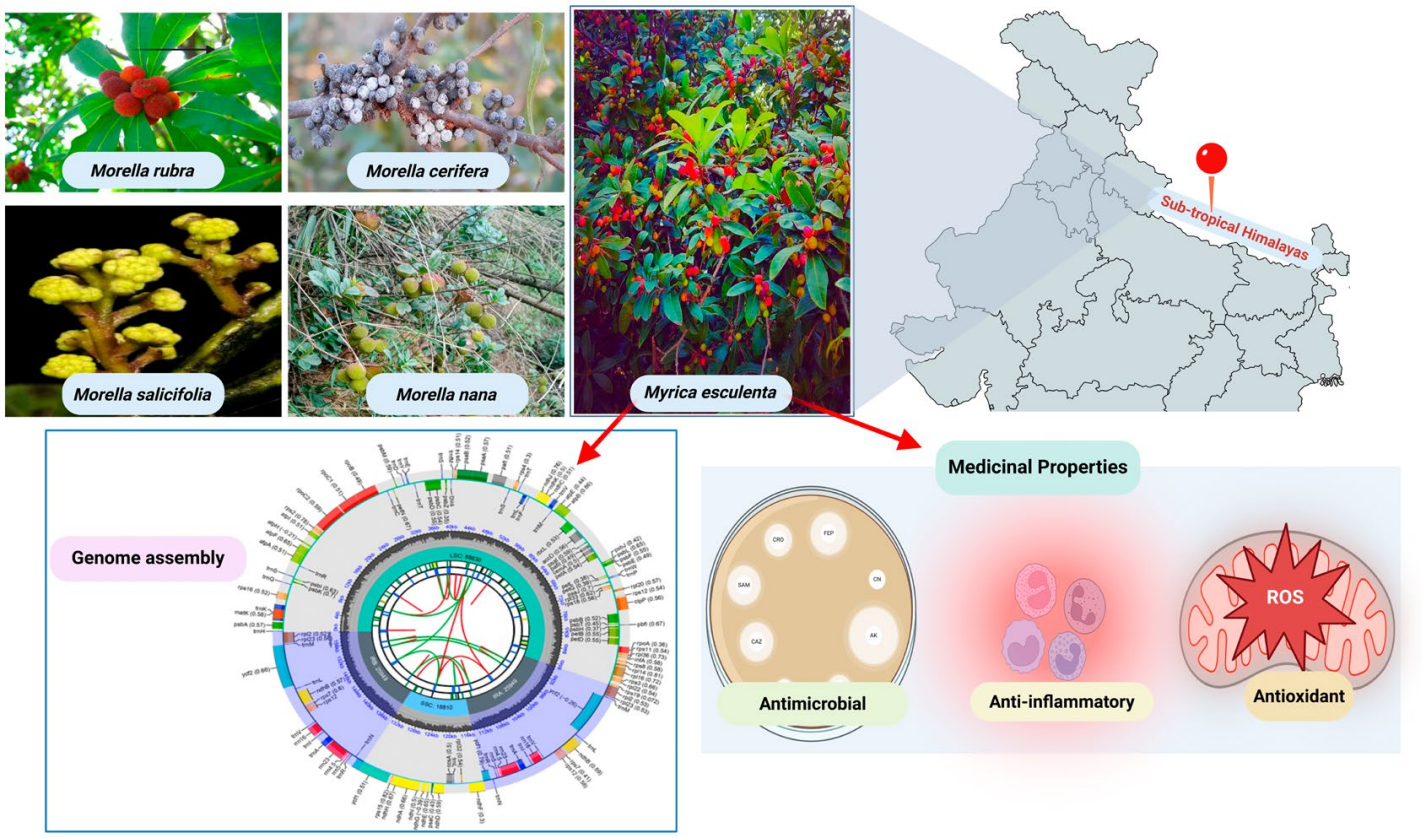
Overview of *Morella* and *Myrica* species with emphasis on *Myrica esculenta*.

Central to this phylogenetic investigation is the chloroplast, a double‐membraned organelle that houses independent DNA and is critical for photosynthesis (Mustardy et al. 2008). Originating from an ancient endosymbiotic event involving a photosynthetic bacterium, chloroplasts are characterized by their highly conserved genomic structures, making them indispensable in phylogenetic analysis, species classification, and even the authentication of herbal materials (Guo et al. 2022). The angiosperm chloroplast genome typically features a quadripartite structure consisting of a large single‐copy (LSC) region, a small single‐copy (SSC) region, and two inverted repeats (IRs) (Oldenburg and Bendich 2016; Maheswari et al. 2021). Given their evolutionary significance and stability, chloroplast genomes are crucial for the molecular taxonomy and evolutionary biology studies within the plant kingdom (Daniell et al. 2016; Singh et al. 2021). Figure 1 is an overview of *Morella* and *Myrica* species with emphasis on *Myrica esculenta*.

*Myrica esculenta* plays an essential ecological role in the Himalayan ecosystem as an evergreen tree that thrives in subtropical and temperate forest habitats. It supports local biodiversity by providing fruit and shelter for various birds and mammals. Its presence is also associated with soil stabilization and microclimatic regulation in hilly terrains. Understanding its chloroplast genome contributes to broader insights into how plants adapt to high‐altitude and stress‐prone environments, offering evolutionary clues valuable for conservation biology and biodiversity management. Recent research by Liu et al. (2017) on *Myrica* species, including *Myrica rubra* , highlighted the utility of complete chloroplast genomes in comparative analyses within the Fagales. However, the chloroplast genome of *Myrica esculenta* remains unreported in the NCBI Organellar Genome Resource to date (NCBI 2024). This study aims to fill this gap by presenting the first assembly of the complete chloroplast genome of *M. esculenta* , thus providing a foundational resource for future investigations into the evolution and biodiversity of both the *Myrica* species and the wider Myricaceae family.

## 2 Materials and Methodology

### 2.1 Genome Data Acquisition and Assembly

Whole genome sequencing (WGS) raw data for *Myrica esculenta* were obtained from the NCBI Sequence Read Archive (SRA) database, specifically under the GenBank accession number SRR11309743. The data, comprising paired‐end reads, were downloaded using the fastq‐dump tool from the SRA Toolkit. Additionally, WGS data for comparative analyses with other *Myrica* species were sourced from the European Nucleotide Archive (ENA) database (https://www.ncbi.nlm.nih.gov/sra).

Chloroplast genomes were assembled using both de novo and reference‐based strategies. The de novo assembly was performed using NovoPlasty v.4.3.2 (Dierckxsens et al. 2017), and the reference‐based assembly utilized GetOrganelle v1.7.7.0 (Jin et al. 2020). For both methods, the ribulose‐1,5‐bisphosphate carboxylase/oxygenase (*rbc*L) gene sequence of *Myrica salicifolia* (GenBank accession no. MK310272.1) served as the seed sequence.

### 2.2 Genome Annotation and Visualization

The assembled chloroplast genome of *M. esculenta* was annotated using the GeSeq tool (Tillich et al. 2017). Transfer RNAs (tRNAs) were specifically validated using tRNAscan‐SE 2.0 (Lowe and Chan 2016). Visualization of the chloroplast genome map was facilitated by the Chloroplot tool (https://irscope.shinyapps.io/chloroplot/) (Zheng et al. 2020), and the structural organization of intron‐containing genes was highlighted using CPGView (www.1kmpg.cn/cpgview/) (Liu et al. 2023). The comparison of the LSC/IRB/SSC/IRA junctions among these related species was visualized by IRscope (http://irscope.shinapps.io/irapp/) (Amiryousefi et al. 2018), based on the annotations of their available cp genomes in GenBank.

### 2.3 Comparative Genomics and Phylogenetic Analysis

Raw reads were subjected to quality assessment using FastQC, and trimming of adapters and low‐quality sequences was performed using Trimmomatic. For comparative genomic analysis, the de novo assembled chloroplast genome of *M. esculenta* was aligned with four previously reported chloroplast genomes of *Myrica* species, including *Myrica rubra* , *Myrica cerifera* , *Myrica salicifolia*, and *Myrica nana* (GenBank IDs: KY476635.1, MT872488.1, MK310272.1, PQ165046.1, respectively), along with eight other species from the Fagales order. Protein‐coding genes longer than 1000 bp were selected for nucleotide diversity (*Pi*) analysis to ensure robust verbality estimation. Alignments were executed using MAFFT v7.4.0.9 (https://mafft.cbrc.jp/alignment/software/index.html) with default parameters (Katoh and Standley 2013). Post‐alignment, the sequences were trimmed for uniform length. Nucleotide diversity among the chloroplast genomes was calculated using DnaSP v6.0, focusing only on protein‐coding genes exceeding 1000 bp (Rozas et al. 2017). Phylogenetic trees were constructed using the Maximum‐Likelihood method under the GTR + G model in MEGA11, supported by 1000 bootstrap replications (Tamura et al. 2021), and a comprehensive phylogenetic tree was generated to elucidate the evolutionary relationships within the *Myrica* species and between other members of the Fagales order.

## 3 Results and Discussion

### 3.1 Genomic Organization and Mapping of the *M. esculenta* Chloroplast Genome

**FIGURE 2:**
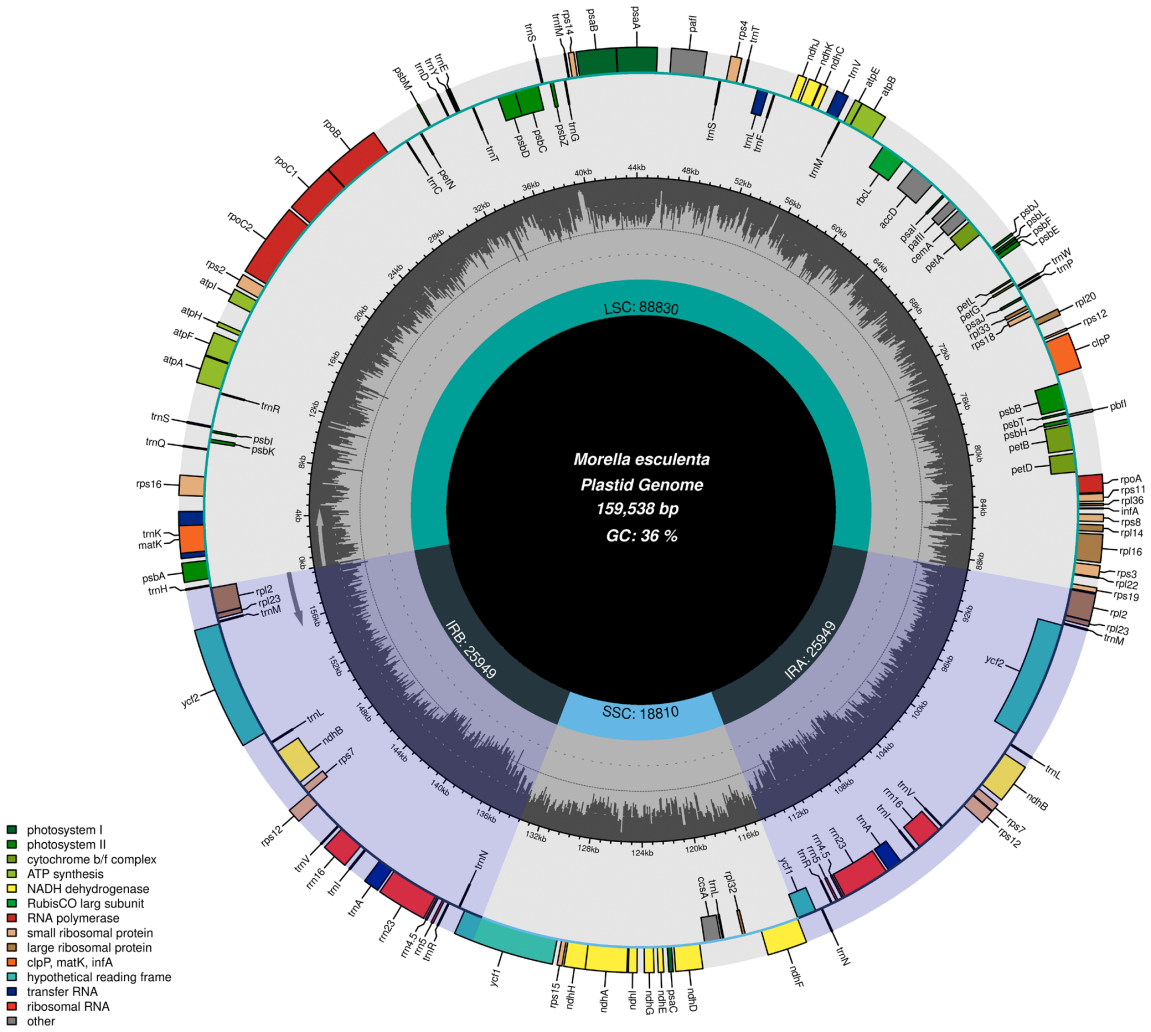
Chloroplast genome circular map of *M. esculenta* .

The complete chloroplast genome of *M. esculenta* spans 159,538 base pairs (bp) and adheres to the typical quadripartite structure found in angiosperms. This structure comprises a large single copy (LSC) region of 88,830 bp, a small single copy (SSC) region of 18,810 bp, and two identical inverted repeat regions (IRs) each of 25,949 bp. Figure 2 illustrates this arrangement and shows the distribution of various gene types, including protein‐coding genes, tRNAs, and rRNAs across these regions. The LSC and SSC regions primarily house genes involved in photosynthesis and other essential metabolic functions, while the IRs include genes responsible for the structural and functional stability of the chloroplast genome.

#### 3.1.1 Functional Implications of Chloroplast Genome Organization

The intricate arrangement of the *M. esculenta* chloroplast genome highlighted in Figure 2, with its clearly delineated IR, LSC, and SSC regions, suggests a highly evolved mechanism for optimizing gene accessibility and expression. The localization of essential photosynthetic genes within the LSC and SSC regions may enhance the efficiency of photosynthesis, while the conservation of structural genes within the IRs could contribute to genome stability and resilience against mutations (Maheswari et al. 2021).

#### 3.1.2 Evolutionary Insights

The comprehensive annotation and mapping of the *M. esculenta* chloroplast genome provide a foundational platform for phylogenetic studies, as illustrated by the gene synteny and conservation patterns observed in Figure 2. Comparative genomic analyses using these data can help elucidate evolutionary relationships within the Myricaceae family and possibly reveal evolutionary strategies that have enabled *M. esculenta* to adapt to its specific ecological niches (Cheng et al. 2020).

### 3.2 Gene Content and Introns

**FIGURE 3:**
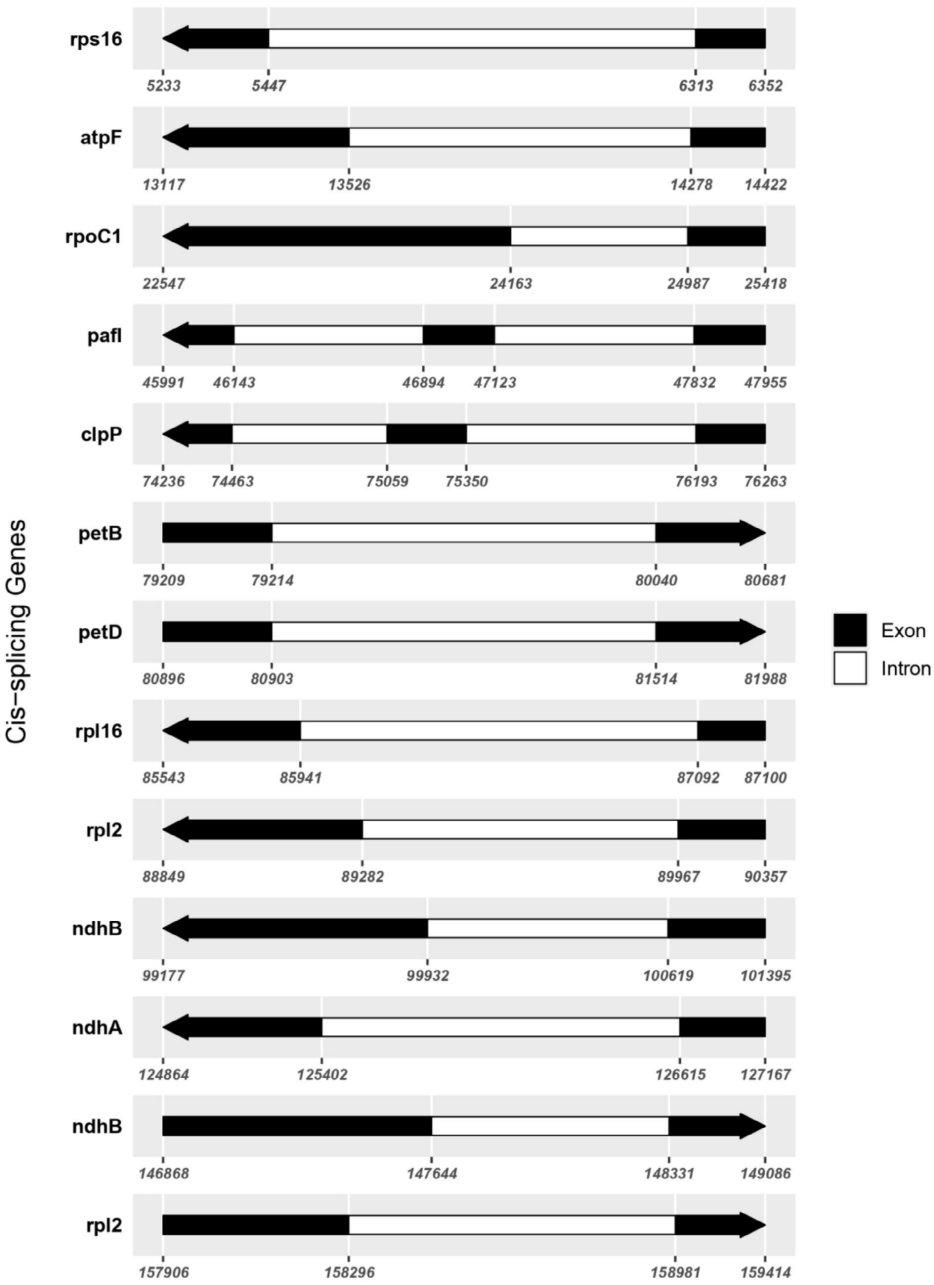
Details of intron‐containing genes (cis‐splicing genes) trans‐splicing genes of cp genome of *M. esculenta*.

The chloroplast genome contains 130 unique genes, which include 86 protein‐coding genes, 36 tRNA genes, and 8 rRNA genes. Intriguingly, 11 of the protein‐coding genes contain one intron, whereas two genes, *clp*P and *ycf3*, harbor two introns each, potentially indicating a complex regulation mechanism that may affect gene expression efficiency and protein assembly. Figure 3 provides a detailed map of these genes, showing the exact positions of exons and introns, which is crucial for understanding the post‐transcriptional modifications and gene expression regulation within the chloroplast.

#### 3.2.1 Role of Introns in Gene Expression

The detailed exon‐intron mapping provided in Figure 3 demonstrates the complexity of the chloroplast genome's transcriptional landscape. Introns in chloroplast genes, like those observed in *clp*P and *ycf3*, are known to play crucial roles in the regulation of gene expression. They can affect mRNA splicing and stability, potentially enhancing the adaptability and functionality of chloroplast proteins under various environmental conditions (Daniell et al. 2016). The presence of these introns, especially in genes involved in essential metabolic pathways, underscores their possible adaptive significance in maintaining cellular function and resilience.

The detailed genomic mapping and analysis of the chloroplast genome of *Myrica esculenta* not only enhances our understanding of the molecular architecture and functional dynamics of this essential organelle but also sets the stage for future studies focusing on the evolutionary biology and conservation of this and related species. The GenBank submission of this genome under accession number PQ046282.1 ensures that these valuable data will contribute to broader research efforts in plant science and phylogenetics.

### 3.3 Nucleotide Diversity (*Pi*) Among *Myrica* Species

**FIGURE 4:**
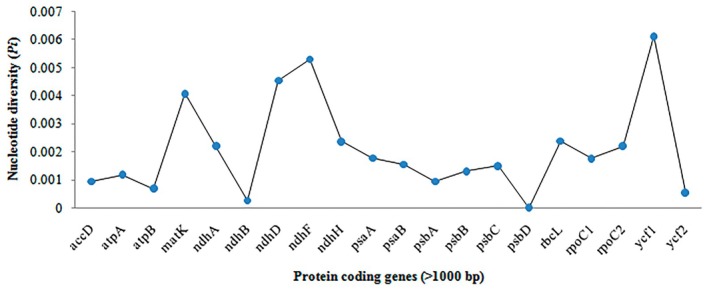
The nucleotide polymorphism for chloroplast genomes of *Myrica* species was calculated using DnaSP 6.0. The protein‐coding genes were selected that are more than 1000 bp in size. Three of the most divergent regions (*ycf1*, *ndh*F, *ndh*D, and *mat*K) are suggested as mutational hotspots.

Nucleotide diversity (*Pi*) analysis among the chloroplast genomes of five *Myrica* species has identified several regions of high genetic variability. Figure 4 illustrates the *Pi* values across various protein‐coding genes longer than 1000 bp. Notably, the *ycf1* gene exhibits the highest diversity with a *Pi* value of 0.00613, followed by *ndhF* (0.0053), *ndhD* (0.00454), and *matK* (0.00408). These values indicate significant genetic differentiation within these regions, suggesting their potential utility in species discrimination and phylogenetic studies.

#### 3.3.1 Implications of High Nucleotide Diversity in *Myrica* Chloroplast Genomes

The observed nucleotide diversity in the chloroplast genomes of *Myrica* species, particularly in the *ycf1*, *ndhF*, *ndhD*, and *matK* genes, underscores their potential as molecular markers for species identification and phylogenetic analyses. The *ycf1* gene, with the highest *Pi* value, stands out as a particularly effective marker. This gene's significant variability, as highlighted in Figure 3, could be advantageous for resolving phylogenetic relationships within the genus *Myrica*, which is crucial for accurate species identification and authentication in both ecological studies and commercial applications. A comparative analysis of nucleotide diversity shows that *ycf1* (*Pi* = 0.00613) outperforms traditional barcode genes such as *matK* (0.00408), *ndhF* (0.0053), and *rbcL* (0.00195) in variability, making it a promising candidate for species‐level discrimination. Standard barcoding markers like *rbcL* and *matK*, although widely used, often lack sufficient resolution within certain plant clades such as Fagales (Vassou et al. 2016). Our results align with prior barcoding studies that recommend *ycf1* as a high‐resolution, universal marker.

#### 3.3.2 Utility of Nucleotide Diversity in Conservation and Taxonomy

The regions exhibiting high nucleotide diversity are critical not only for distinguishing between species but also for understanding the evolutionary dynamics within the *Myrica* genus. These hotspot regions, by reflecting the genetic diversity, offer insights into the evolutionary pressures and historical population dynamics of these species. The statistical significance of the diversity in the *ycf1* gene (*p* < 0.0001) further validates its relevance in molecular taxonomy and conservation genetics. Given the identified nucleotide diversity hotspots, future research could focus on developing these segments into robust, species‐specific genetic markers. These markers could be utilized in DNA barcoding, which is essential for the authentication of herbal products derived from *Myrica* species. Additionally, extensive sampling across the genus could help refine these markers and improve their efficacy in phylogenetic analysis and species identification. Studying the differences in nucleotides in the chloroplast genomes of Myrica species is important for creating genetic tools needed for classifying and protecting these plants (Akhunov et al. 2010). The high‐resolution regions identified in this study hold promise for enhancing our understanding of species relationships and diversity within this genus, offering significant implications for both scientific research and practical applications in biodiversity management.

### 3.4 Nucleotide Diversity in *M. esculenta*

**FIGURE 5:**
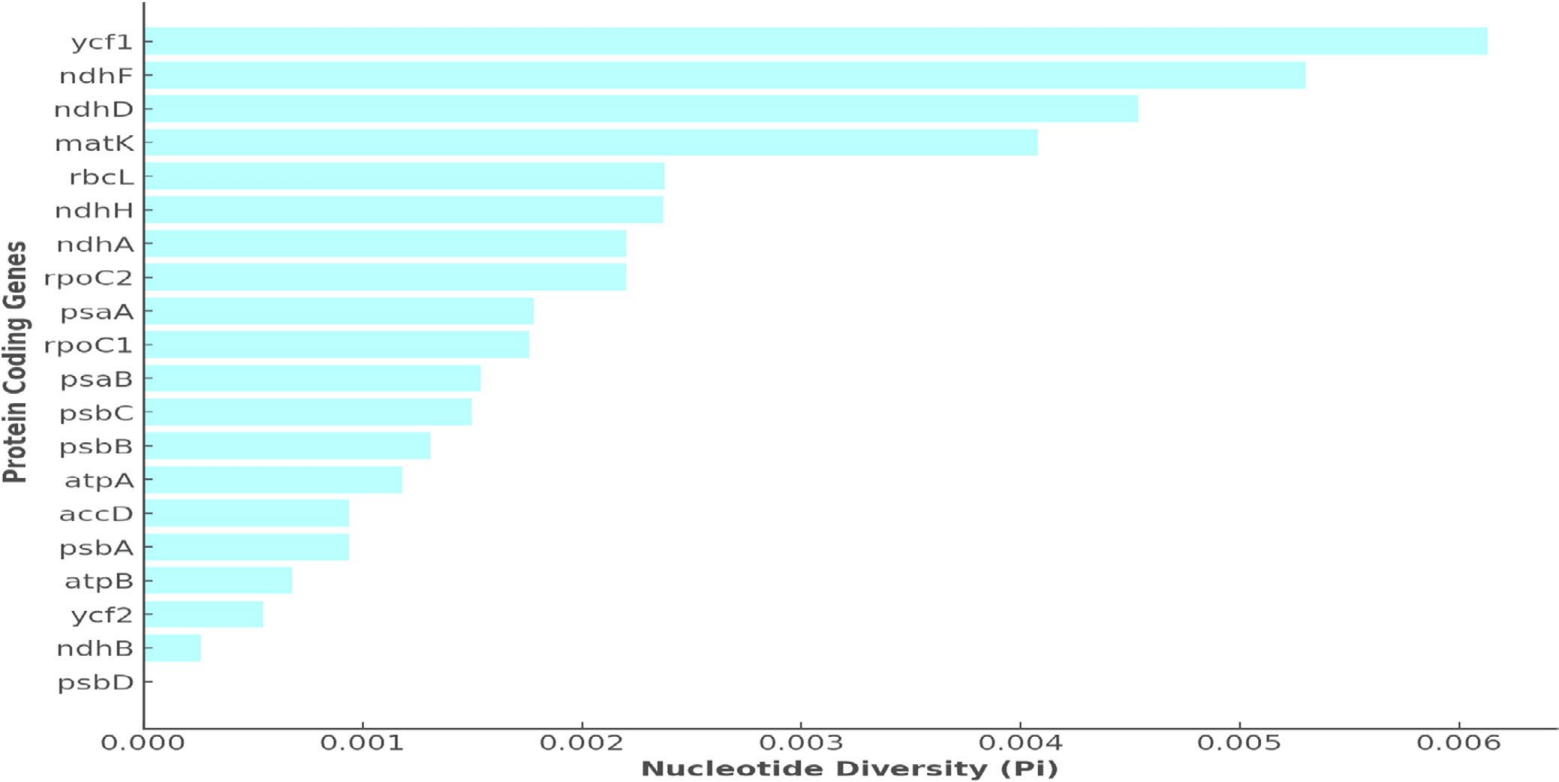
Nucleotide diversity in *M. esculenta*.

The nucleotide diversity (*Pi*) in the chloroplast genome of *M. esculenta* exhibits significant variation among different protein‐coding genes, as depicted in the graph. Notably, the *ycf1* gene shows the highest diversity with a *Pi* value just above 0.006, indicating it has the greatest variation among the sequences analyzed. This is followed by the *ndhF* and *ndhD* genes, which also show relatively high diversity, with *Pi* values above 0.004 and near 0.005, respectively. The gene *matK* also exhibits notable diversity, with a *Pi* value close to 0.004. These genes, particularly *ycf1*, *ndhF*, *ndhD*, and *matK*, could be considered potential markers for phylogenetic studies and species identification within the genus due to their high nucleotide diversity. This variability may reflect adaptation to different environmental conditions or evolutionary pressures, indicating areas of the genome that are more prone to mutations. A lower diversity is observed in other protein‐coding genes such as *psbD*, *ndhB*, and *ycf2*, with *Pi* values significantly lower, suggesting these regions are more conserved across different individuals of *M. esculenta* . Conserved regions often play crucial roles in fundamental cellular processes and thus exhibit less variability (Figure 5).

The variation in nucleotide diversity across these genes provides insights into the evolutionary dynamics of the chloroplast genome and might influence studies focusing on the adaptive evolution, conservation genetics, and systematics of *M. esculenta* . The higher diversity in genes like *ycf1* and *ndhF* might be leveraged to explore genetic drift, gene flow, and selection pressure in this species, offering a genetic window into its ecological adaptations and evolutionary history.

### 3.5 Inverted Repeats of *Myrica* Species

**FIGURE 6:**
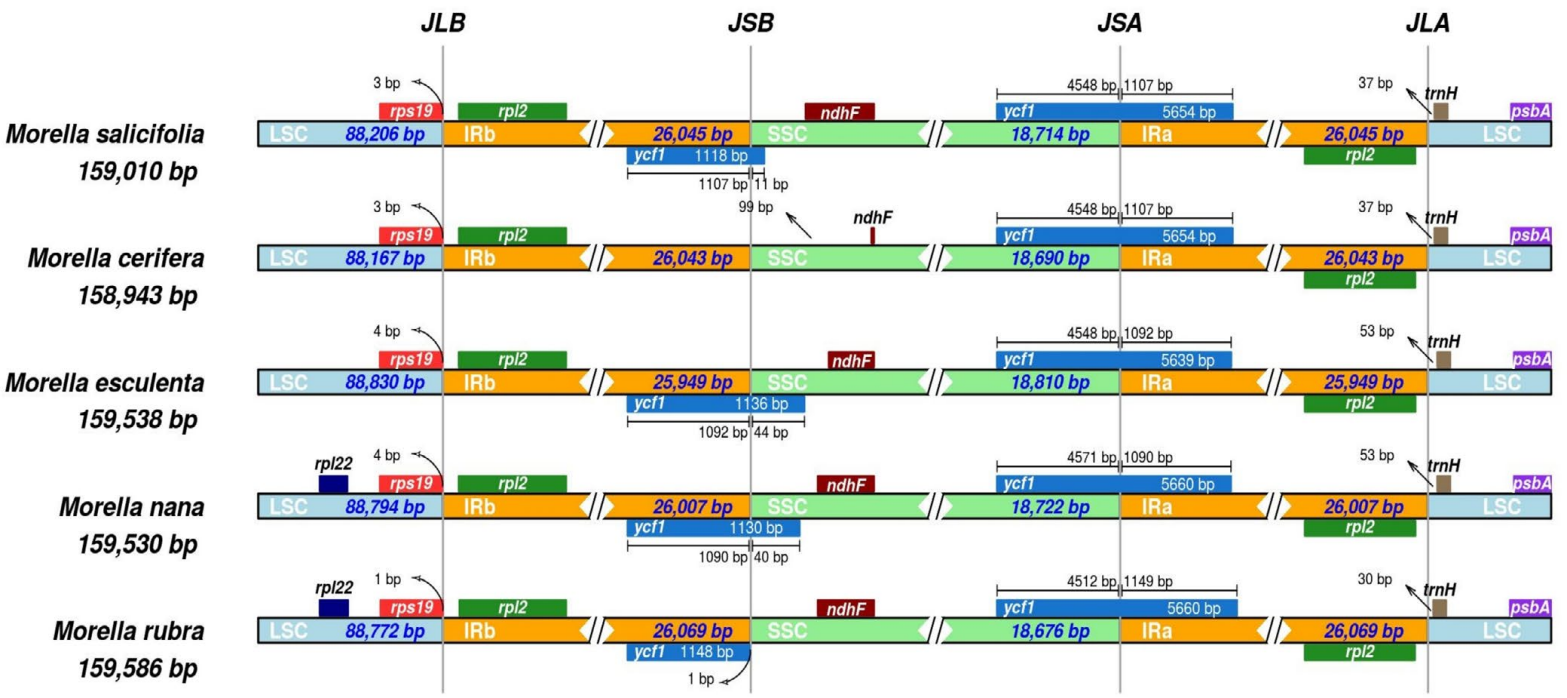
Comparisons of the border region among the chloroplast genomes of five *Myrica* species.

The five *Myrica* chloroplast genomes exhibited high similarity at the LSC/IR/SSC boundaries (Figure 6). In the process of cp genome evolution in angiosperms, the amplification/contraction of the IR boundary and gene loss were considered to be the main reasons for the difference in chloroplast genome size among different species, while the highly variable genes in the IR boundary could be used as evolutionary markers to study the phylogenetic relationship between groups. The *rps19* gene crossed the LSC/IRB (JLB) region with no variation in sequence length within the two parts. The SSC/IRB (JSB) junction occurred between the *ycf1*_like (incompletely duplicated in IRB), except in *M. cerifera* , and the 3′ end of the ndhF gene, with the sequence length of the *ycf1*_like gene within IRB as 1118 to 1148 bp. The *ycf1* gene crossed the SSC/IRA (JSA) region, with 5639 or 5660 bp of *ycf1* within IRA. The *ycf1* related length changes were the only variation detected in these junctions.

### 3.6 Phylogenetic Analysis Based on the *ycf1* Gene

**FIGURE 7:**
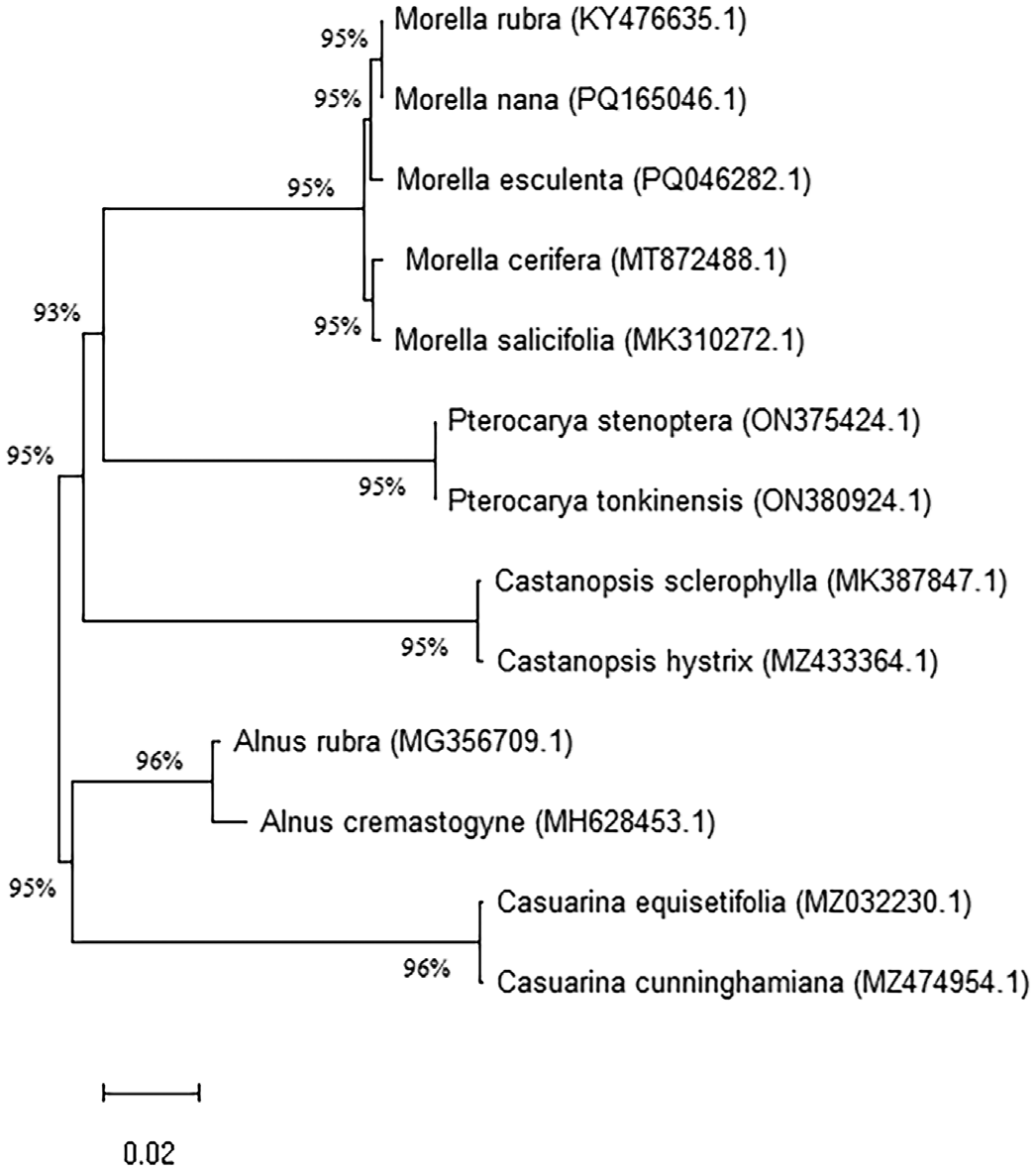
Phylogenetic tree of *M. esculenta* and other related species of Fagales order based on the *ycf1* gene of chloroplast genomes. The numbers on the branches indicate the bootstrap values.

In this study, we constructed a maximum likelihood (ML) phylogenetic tree using the *ycf1* gene sequences from the chloroplast genomes of 13 species within the Fagales order, including five species from the *Myrica* genus. These sequences were aligned using MAFFT with its default settings, ensuring a standardized approach for comparative genetic analysis. The phylogenetic analysis was conducted using the General Time Reversible model plus Gamma distribution (GTR + G) in MEGA software, supported by 1000 bootstrap replicates to assess the robustness of the tree (Figure 7).

The resulting phylogenetic tree revealed that *M. esculenta* is more closely related to *M. rubra* and *M. nana* , while *M. cerifera* and *M. salicifolia* formed a distinct sister clade. This topology indicates a clear divergence within the *Myrica* genus, suggesting evolutionary pathways that might be influenced by geographical separation or ecological niche specialization. The high bootstrap values observed for the clades within *Myrica* imply a strong phylogenetic signal and reliability in the evolutionary relationships presented.

Despite the robustness of the phylogenetic tree obtained, it is important to note that the limited number of complete chloroplast genomes available for the *Myrica* genus may restrict our understanding of the full evolutionary breadth of these species. The current dataset underscores the need for more comprehensive genomic resources to enhance our phylogenetic resolution and accuracy. The complete chloroplast genome of *M. esculenta* , determined in this study, not only enriches our genetic database but also provides crucial insights into the evolutionary dynamics within *Myrica* and the broader Fagales family. These findings have significant implications for biodiversity studies and conservation strategies, particularly for economically and medicinally important species within the order. Further research should aim to expand the chloroplast genome datasets, enabling more detailed phylogenetic frameworks that could assist in unraveling the evolutionary history and ecological adaptations of the Fagales.

## 4 Discussion

In the study of evolutionary relationships within the *Myrica* genus, particularly *M. esculenta* , the use of the *ycf1* gene from the chloroplast genome as a molecular marker has provided significant insights. The *ycf1* gene, known for its high nucleotide diversity, serves as an effective tool for distinguishing species within the genus. Our phylogenetic analysis, constructed using the maximum likelihood method based on the *ycf1* gene sequence, included 13 species from the Fagales order, enhancing our understanding of the genetic connections and divergences among these species.

The phylogenetic tree derived from this analysis reveals that *M. esculenta* shares a closer evolutionary relationship with *M. rubra* and *M. nana* than with *M. cerifera* and *M. salicifolia* , which form a separate sister clade. This alignment suggests that despite the close geographical and ecological proximities, significant genetic differentiation has occurred within the *Myrica* genus. Such differentiation could be attributed to isolated evolutionary paths driven by distinct ecological pressures or minor genetic drifts accumulating significant differences over time. The distinct clades observed in our phylogenetic tree may correspond to biogeographical separations within the *Myrica* species across Asia. The divergence between *M. esculenta* and *M. cerifera/salicifolia* could reflect evolutionary adaptation to distinct ecological niches in the Himalayan versus North American or coastal habitats. Similar evolutionary patterns based on *ycf1* have also been noted in Lamiaceae (Balaji and Parani 2024), supporting its utility in resolving lineage diversification. Moreover, the strong bootstrap support for the clades within *Myrica* indicates that the phylogenetic tree is robust, providing a reliable depiction of the evolutionary lineage. However, it is crucial to acknowledge that the limited number of complete chloroplast genomes currently available poses a challenge in fully resolving the phylogenetic relationships of *Myrica* species. As such, our study highlights the need for further genomic sequencing across the genus to improve the resolution and breadth of our phylogenetic understanding.

Our results contribute significantly to the field of plant systematics, particularly within the Fagales order, by confirming the utility of the *ycf1* gene as a molecular marker in phylogenetic studies and species identification. They underscore the potential of chloroplast genome analysis not only in understanding the evolutionary history of taxa but also in aiding in the conservation and management of biodiversity (Liu et al. 2019). This study lays a foundational framework for future research aimed at expanding the chloroplast genome databases, thereby facilitating more comprehensive evolutionary studies within the *Myrica* genus and beyond.

## 5 Conclusion

This study has successfully elucidated the phylogenetic relationships within the *Myrica* genus, particularly focusing on *Myrica esculenta*, using the chloroplast *ycf1* gene as a molecular marker. The comprehensive analysis of the chloroplast genome has highlighted the *ycf1* gene's high nucleotide diversity, establishing its utility in distinguishing species within the *Myrica* genus and potentially other members of the Fagales order. Our findings reveal that *M. esculenta* is closely related to *M. rubra* and *M. nana* , while it forms distinct genetic lineages from *M. cerifera* and *M. salicifolia* . These results not only add to our understanding of the genetic structure and evolutionary dynamics of *Myrica* species but also contribute to broader phylogenetic studies within the Fagales. The phylogenetic tree, supported by robust bootstrap values, underscores the reliability of the *ycf1* gene in resolving complex evolutionary relationships. However, the study also acknowledges the limitations posed by the availability of complete chloroplast genome sequences within the genus. The current dataset, though informative, highlights the need for more extensive genomic data to enhance phylogenetic resolution and accuracy.

As we advance, it is imperative to continue collecting and sequencing chloroplast genomes from a wider array of species within the *Myrica* genus and the Fagales order. This will not only solidify our current findings but also uncover finer details of evolutionary history, aiding in conservation efforts and the sustainable use of these plants, many of which hold significant economic and medicinal value. Ultimately, the insights gained from this study serve as a valuable resource for future research aimed at understanding the evolution and biodiversity of the Fagales family. By expanding our genomic databases and applying rigorous phylogenetic methodologies, we can further explore the intricate genetic tapestry of this group, providing critical information for taxonomy, conservation, and the sustainable exploitation of these species.

## Author Contributions

**Raju Balaji:** conceptualization (equal), data curation (equal), investigation (equal), writing – original draft (equal). **Subbalakshmi Easwaran:** conceptualization (equal), formal analysis (equal), investigation (equal), writing – review and editing (equal). **Krishnamoorthy Devanathan:** formal analysis (equal), methodology (equal), validation (equal), writing – review and editing (equal). **Swati Sharma:** formal analysis (equal), investigation (equal), validation (equal), writing – review and editing (equal). **Taha Alqahtani:** data curation (equal), formal analysis (equal), validation (equal), writing – review and editing (equal). **Daniel E. Uti:** conceptualization (equal), validation (equal), writing – original draft (equal), writing – review and editing (equal). **Tabarak Malik:** conceptualization (equal), investigation (equal), validation (equal), writing – review and editing (equal).

## Conflicts of Interest

The authors declare no conflicts of interest.

## Data Availability

The data that support the findings of this study are openly available in NCBI (https://www.ncbi.nlm.nih.gov/). The complete chloroplast genome of Garcinia indica was deposited in GenBank under the accession PQ046282.1 (https://www.ncbi.nlm.nih.gov/nuccore/PQ046282.1). The associated NGS sequencing data files are publicly available from the SRA under the accession numbers SRR11309743. Data are contained within the article and supplementary materials.
